# A Novel Berberine–Glycyrrhizic Acid Complex Formulation Enhanced the Prevention Effect to Doxorubicin-Induced Cardiotoxicity by Pharmacokinetic Modulation of Berberine in Rats

**DOI:** 10.3389/fphar.2022.891829

**Published:** 2022-07-22

**Authors:** Shichang Zhang, Yiwei Zhao, Liangjun Tan, Sheng Wu, Qing Zhang, Boxin Zhao, Guofeng Li

**Affiliations:** ^1^ Department of Pharmacy, Nanfang Hospital, Southern Medical University, Guangzhou, China; ^2^ Rational Medication Evaluation and Drug Delivery Technology Lab, Nanfang Hospital, Southern Medical University, Guangzhou, China; ^3^ Guangdong Provincial Key Laboratory of New Drug Screening, Southern Medical University, Guangzhou, China

**Keywords:** berberine, glycyrrhizic acid, pharmacokinetics, DOX-induced cardiotoxicity, complex

## Abstract

Developing a new drug delivery system is one of the useful approaches to overcome the limited use of berberine (BBR) to enhance its absorption and bioavailability. We prepared a novel berberine–glycyrrhizic acid (BBR–GL) complex formulation to increase the plasma concentration and bioavailability of BBR by improving BBR solubility and lowering the absorption barrier. The complex formulation with BBR and GL in the ratio 1:1 was developed through the self-assembly process and evaluated *in vitro*. Compared with BBR and BBR/GL physical mixture, the BBR–GL complex showed different characteristics by SEM, DSC, FT-IR, and PXRD measurement. In pharmacokinetic evaluation, the BBR–GL complex significantly increased the plasma concentration of BBR and the major metabolite berberrubine (BBB), with the AUC of BBR elevated to 4.43-folds, while the complex was safe as BBR. Furthermore, doxorubicin (DOX) was used to induce cardiotoxicity. Hematological study, histopathological examinations, electrocardiography (ECG), cardiac secretion measurement, and biochemical index analysis proved that the model of doxorubicin-induced cardiotoxicity (DIC) was conducted successfully. With the AUC of BBR increasing in the BBR–GL complex and the absorbed complex itself, the BBR–GL complex enhanced prevention effect to DIC and exhibited a significant prevention effect to attenuate heart damage. Our findings demonstrated that a novel BBR-loaded BBR–GL complex formulation could increase BBR plasma concentration. Improvement of BBR bioavailability by the BBR–GL complex could coordinate with GL to attenuate DIC. Concerning the safety of the drug delivery system at present, the BBR–GL complex could be a potential therapeutic formulation for the prevention of cardiac damage in the clinical application of doxorubicin.

## 1 Introduction

Berberine is one of the most important isoquinoline alkaloid isolated and has been used for the treatment of bacteria-associated diarrhea and other gastrointestinal infections in China (Li L. et al., 2020). It was found in many traditional Chinese medicinal herbs, such as *Coptis chinensis* Franch (Chen et al., 2008), *Coptis japonica* (Thunb.) Makino (Min et al., 2006), and *Phellodendron chinense* C. K. Schneid (Liu Y. et al., 2010). It has been used for thousands of years and exhibited remarkable efficacy for dispelling wind, clearing heat, and removing toxicity.

As a natural product, berberine has been found to have several pharmacological effects on anti-inflammation (Pei et al., 2019; Liu et al., 2020), antihypertension (Suadoni and Atherton, 2021), and anticancer (Majidzadeh et al., 2020) and has efficacy for diabetes (Li W. et al., 2020). In spite of these potential pharmacological effects, the major obstacles for its therapeutic application are its poor solubility and low bioavailability. Liu et al. first found that extensive elimination in the small intestine and high hepatic extraction were the major PK causes that resulted in very low plasma concentrations of berberine in rats, and berberine’s AUC value post intraduodenal administration was nearly 0.5% of its AUC value post intraportal vein administration. These results suggested that first-pass elimination occurred predominantly in the rat intestine (Liu YT. et al., 2010). Moreover, current pharmacokinetic studies in animals of berberine have some contradictions. Thus the *in vitro* and *in vivo* performance results in animals may not exhibit the same pharmacological effect because of the pharmacokinetic diversity. Although few studies about the solubilization of berberine by surfactant and other nano preparation using PEG (Sujitha et al., 2020) and liposome (Allijn et al., 2017) were reported, safety and stability were the main problems in its clinical use.

Similar to Coptis being a commonly used crude medicine, Glycyrrhizae radix is also frequently used in traditional Chinese medicine (TCM) formulas, which is known in the theory of “ten prescriptions nine grass.” Glycyrrhizic acid (GL), the major active ingredient of Glycyrrhizae radix, shows diverse pharmacological capacity in deintoxication, anti-inflammation, and liver protection (Baltina, 2003; Nose et al., 2017). Besides, Glycyrrhizae radix is commonly combined with *Coptis chinensis*, Baikal skullcap, and *Phellodendron amurense* as the TCM composing principle. Unfortunately, the interaction of glycyrrhizic acid and berberine in pharmacology and pharmaceutics is still unclear.

GL can form complexes or micelles in different conditions due to its amphiphilic structure (Polyakov et al., 2006). Pharmacologically, GL is frequently used as a supplement or solubilizer coadministrated with hydrophobic drugs to improve their efficacy and reduce toxicity (Lu et al., 2018; Zhang et al., 2018). In our previous study, GL has been proved to be used as a drug carrier of some sparingly soluble drugs such as paclitaxel (Yang et al., 2015), podophyllotoxin (Wang et al., 2016), and hydroxycamtothecin (Cai et al., 2019). All these studies showed GL had strong solubilization capability to some sparingly soluble compounds. But there are no reports on whether GL could enhance the solubility of BBR or increase the bioavailability.

Besides, in a recent study, we found glycyrrhizin improved autophagy flux *via* HMGB1-dependent Akt/mTOR signaling pathway that contributed to attenuating doxorubicin-induced cardiotoxicity (DIC). It was a novel insight into the underlying mechanisms of cardioprotective action of GL and reported GL could be a potential candidate for the prevention of DIC (Lv et al., 2020).

Hence, our present work aimed to prepare a novel berberine-glycyrrhizic acid (BBR-GL) complex formulation to increase the solubility of BBR. Additionally, the pharmacokinetics of berberine and its major metabolites in rats were evaluated to investigate the bioavailability improvement, to construct a new BBR–GL complex system used for synergistic prevention of DIC.

## 2 Materials and Methods

### 2.1 Drugs and Chemicals

Berberine hydrochloride (BBR, purity>98%) and GL (purity>98%, HPLC grade) were purchased from Shanghai Macklin Biochemical Co., Ltd. (Shanghai, China). GL (purity>98%) was purchased from Shanghai Yuanye Biotechnology Co. BBR (purity = 99%, HPLC grade) was obtained from National Institutes for Food and Drug Control (Beijing, China). Sodium hydroxide (NaOH, AR) was obtained from Guangdong Guanghua Sci-Tech Co., Ltd. (Swatow, China).

### 2.2 Animals

Male Sprague Dawley (SD) rats of 180–220 g were purchased from the Laboratory Animal Center, Southern Medical University (SMU, Guangzhou, China; permit number: 44002100030650). All the animals were kept under an automated 12-h light–dark cycle at a controlled temperature of 24 ± 2°C and relative humidity of 50 ± 10% and had free access to dry fodder and tap water. The animal experiment protocol was approved by the Animal Care Ethics Committee of SMU, complying with the regulations for animal experimentation issued by the State Committee of Science and Technology of the People’s Republic of China.

### 2.3 Preparation of the Berberine–Glycyrrhizic Acid Complex

For the molecular ratio of 1:1 BBR:GL, GL (332 mg) was dissolved in 20 ml preheated 50% ethanol, and a solution of BBR (150 mg, dissolved in 30 ml preheated 80% ethanol and added in measured NaOH to remove hydrochloric acid) was added dropwise into the preheated aqueous GL solution. For the molecular ratio of 2:1 BBR:GL, 150 mg BBR was added and 166 mg GL was added. For the molecular ratio of 1:2 BBR:GL, 150 mg BBR was added and 664 mg GL was added. After 1 h mild stirring to form a homogeneous medium, the mixture was then dispersed with an ultrasonic homogenizer for 1 h and filtered through 0.45-μm filters. Powder of BBR-GL complex was obtained after the above-mentioned subsequent filtrate was evaporated by rotary evaporator under vacuum. The physical mixture of BBR and GL was obtained by mixing the powder of BBR and GL.

### 2.4 Solubility Studies

We quantitatively tested the BBR solubility released from BBR single drug and BBR-GL complex (the molecular ratios of BBR:GL were 2:1, 1:1, and 1:2) in PBS with different pHs. The drug powder of BBR and BBR-GL was excessively added in a 5 ml tube with solvent. They were shaken in a shaking incubator at 25°C for 48 h to achieve solubility equilibrium (dos Santos et al., 2011; Cai et al., 2019). The samples of BBR and BBR-GL complex in PBS with different pHs (2, 4, 6, 7.4) were tested and then filtered through 0.45-μm filters. The concentrations were detected by high-performance liquid chromatography (HPLC, 1290, Agilent, Palo Alto, United States).

### 2.5 Characterization of the Berberine–Glycyrrhizic Acid Complex

#### 2.5.1 Morphological and Micelle Size Analysis of Berberine–Glycyrrhizic Acid

Morphological analysis of BBR-GL was done by scanning electron microscopy (SEM). After drying overnight, the powders of BBR, GL, mixture, and BBR-GL complex were sputter-coated with gold. The SEM images of BBR, GL, mixture, and complex were obtained using a Hitachi S-3000N scanning electron microscope (Tokyo, Japan). The drug size distribution was determined *via* Zetasizer Nano ZS (Malvern, England).

#### 2.5.2 Fourier Transform Infrared Spectroscopy

IR (KBr pellet) spectra were recorded on an FT-IR spectrometer Vertex 70 (Bruker, Karlsruher, Germany) to further confirm the cocrystal structure. For each sample, a total of 64 scans were collected over the range of 4,000–400 cm^−1^ with a resolution of 0.2 cm^−1^.

#### 2.5.3 Differential Scanning Calorimetry Measurement

DSC analysis was carried out by DSC214 polymer (Netzsch, Munich, Germany). The samples (10 mg), including BBR, GL, mixture, and complex, were weighed in aluminum pans. The samples were tested under nitrogen atmosphere (40 ml/min) and heated in the range of 25–250°C at a heating rate of 20°C/min.

#### 2.5.4 Powder X-Ray Diffractometry

Powder X-ray diffractometry (PXRD) analysis was performed on an Empyrean advanced diffractometer pattern using Cu Kɑ radiation at room temperature. The diffractometer was operated at 30 kV and 10 mA. Data were collected in the range from 3° to 80° (2θ) with a step size of 0.02 and a dwell time of 2 s per step. For each sample, approximately 60 mg of powder was gently pressed with a silicon slide to obtain a flat powder bed surface before data collection.

### 2.6 *In Vitro* Drug Release Profile and Quantitative Berberine by HPLC

To evaluate the drug release profile, 1 ml solution of BBR and BBR-GL containing 1 mg BBR were placed in a dialysis bag (MW = 3,000 Da) respectively. Then, they were, respectively, immersed into 300 ml of water or PBS (pH = 7.4) at 37°C with gentle shaking (100 r/min). Samples (0.5 ml) were withdrawn at predetermined time intervals and the same volume of fresh medium was compensated. The mixed solution was centrifuged at 14,000 rpm for 10 min and the supernatant was analyzed by HPLC.

Briefly, the BBR concentration was quantitatively determined using an HPLC system (HPLC, 1290, Agilent, Palo Alto, United States) with Ecosil C18 analytical column (5 μm, 4.6 mm × 150 mm) at 40°C. The mobile phase consisted of 0.01 M NH_4_H_2_PO_4_, adjusted to 2.8 pH by adding dropwise phosphoric acid (A) and acetonitrile (B). Samples were eluted at a flow rate of 1 ml/min. Gradient elution was used according to the following schedule: the initial proportion of B was 10%; 20% B for 2−4 min, 50% B for 9−13.9 min, and 10% B for 14−20 min. The total analysis time was 20 min. The detective wavelength was set at 345 nm. The injection volume was 2 μl, and the retention time was approximately 10.4 min. The accuracy, precision values, and recovery rate were up to standard. All of the samples were filtered through a 0.45 μm membrane filter before being detected. *In vitro* drug release data were further analyzed by fitting to zero-order, first-order, and Higuchi models, and regression analysis was performed to obtain the best fitted model.

### 2.7 Pharmacokinetic Studies

#### 2.7.1 Administration of the Berberine–Glycyrrhizic Acid Complex to Sprague Dawley Rats

A total of 24 SD rats were randomly assigned into two groups: oral administration and intravenous injection. For oral administration, the BBR and BBR-GL were dissolved in water. Each rat received BBR and BBR-GL solutions containing the same dose of 40 mg/kg BBR. For intravenous injection (iv), each rat received *via* the tail vein the BBR solution and the BBR-GL complex at a dose of 1 mg/kg BBR, respectively. Each group contained six rats. After fasting for 12 h, blood samples were collected from retro-orbital plexus into heparinized tubes at 5, 15, 30, 60, 120, 360, 720, and 1,440 min and centrifuged immediately at 3,500 rpm for 10 min. The plasma samples were then stored at −20°C until analyzed by LC/MS-MS (Agilent, Palo Alto, United States) analysis.

#### 2.7.2 Sample Preparation

Plasma samples (50 μl) were pipetted into another tube mixed with 100 μl acetonitrile containing tetrahydropalmatine as internal standard (IS). The mixture was centrifuged at 14,000 rpm for 10 min after vortex for 30 s. A 5 μl aliquot of the supernatant was injected into the LC-MS/MS system for analysis.

#### 2.7.3 LC-MS/MS Analysis of Berberine and Its Metabolites

Agilent 6460 Triple Quad LC-MS system (Agilent, Palo Alto, America) coupled with an Agilent 1260 series HPLC system was used for the analysis of berberine and its metabolites using the modified method of Feng et al. (2020).

The separation was performed on an Agilent poroshell EC-C18 column (3.0 mm × 50 mm, 2.7 μm; Agilent, United States) using a gradient elution of the mobile phase that consisted of methanol (A) and water with 0.1% formic acid (B) at a flow rate of 0.5 ml/min. The composition of the mobile phase was as follows: 70% B for 0–0.5 min, 30% B for 1.2–2.5 min, and 70% B for 2.6–5 min. Mass spectra were recorded by electrospray ionization with a positive mode. Quantification was carried out using MRM at *m*/*z* 336.0 → 320.3/292.3 for berberine, *m*/*z* 322.1 → 307.1/279.1 for berberrubine (BBB), and *m*/*z* 356.2 → 192.2/165.2 for tetrahydropalmatine. Calibration was applied on a standard curve in the range of 0.05–100 ng/ml of berberine and BBB, respectively.

### 2.8 Doxorubicin-Induced Cardiotoxicity Model

**FIGURE 1:**
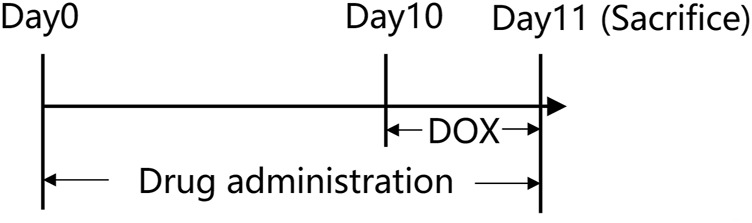
A diagram of the experiment design in rats.

In the current study, 36 rats were randomly separated into the following six groups: control (0.9% saline), DOX treatment (20 mg/kg), DOX plus BBR treatment (40 mg/kg), DOX plus GL treatment (65 mg/kg, equimolar with BBR), DOX plus BBR-GL treatment (105 mg/kg, containing BBR 40 mg/kg), and single BBR-GL treatment (105 mg/kg, containing BBR 40 mg/kg). Each group contained six rats. BBR and BBR-GL were orally administered at the previously mentioned doses once daily for all the 11 days, while the control and DOX groups received equal volumes of 0.9% saline intragastrically. On day 10, all rats, except for the rats in the control group and the single BBR-GL treatment group, received intraperitoneal injections of DOX (20 mg/kg/day diluted with 0.9% saline). After the last administration of drug or saline on day 11, the rats were euthanized for subsequent studies after 12 h. The timeline of the animal administration is illustrated in Figure 1.

### 2.9 Hematological Study and Histopathological Examinations

To analyze the histopathological changes in cardiac tissue, the rats were euthanized with an intraperitoneal injection of 2 ml of pentobarbital. Parts of blood sample for the rats in the control group and the single BBR–GL treatment group were collected and analyzed through auto-analyzer (Cobas C501, Roche, United States) at a pathological laboratory for complete blood count (CBC) analysis. Serum samples were collected from the other part of the blood sample by centrifugation at 4°C, 3,500 rpm for 10 min and the following biochemical indexes were measured: liver function indexes [alanine aminotransferase (ALT), aspartate aminotransferase (AST), bilirubin total (TBIL)] and kidney function indexes [uric acid (UA), urea formaldehyde (UREA), and creatinine (CRE)]. All the indexes were measured using Automatic Chemistry Analyzer (BC-6600, Mindray Bio-Medical Electronics Co., Ltd., Shenzhen, China).

Except for the single BBR-GL treatment group, the heart was excised, and one part of the myocardium was fixed overnight in 10% formalin, embedded in paraffin, and dehydrated in an ascending series of ethanol (70, 80, 90, and 100%). The tissue samples were embedded in paraffin wax and cut into 5-μm-thick sections. The sections were mounted on normal glass slides and stained with hematoxylin and eosin (HE) for 2 min for the histological examination. The specimens were examined under a light microscope (magnification, ×200, Olympus BX-51; Olympus Corporation, Tokyo, Japan).

### 2.10 Electrocardiography, Cardiac Secretion Measurement, and Biochemical Index Analysis

The rats were anesthetized and fixed on a table in the supine position using an ECG recorder (AD INSTRUMENTS, Australia). Subcutaneous needle electrodes were connected to the rats for the limb lead at position II, and electrocardiograms were recorded. The blood sample was collected from abdominal aorta and immediately centrifuged at 3,500 rpm for 10 min. The levels of BNP, NT-proBNP, cTnI, and cTnT released into the rat plasma were measured using ELISA kits from Jiangsu Meimian Industrial Co., Ltd. The fresh heart tissue of the DIC model was collected, excised, weighted, frozen in liquid nitrogen, and then stored at −80°C. The tissue was rinsed, homogenized, and centrifuged at 5,000 rpm at 4°C for 15 min; then the levels of SOD and MDA in the supernatant were detected using the corresponding detection kits.

### 2.11 Data Analysis and Statistical Analysis

The pharmacokinetic parameters were calculated by noncompartment analysis using the WinNonlin version 8.2 software (Pharsight, Certara, NJ, United States). All data for statistical analysis were presented as mean value and standard deviation (mean ± SD) for all experiments with at least three replicates. Statistical analysis was performed using SPSS for Windows (version 25.0; IBM Corp., Armonk, NY, United States). The results were analyzed by Student’s *t*-test for two groups or one-way ANOVA for multiple groups with Tukey *post hoc* test. Results were considered to be significant when *p* < 0.05.

## 3 Result

### 3.1 Glycyrrhizic Acid Could Enhance the Solubility of Berberine by the Berberine–Glycyrrhizic Acid Complex Form

**FIGURE 2:**
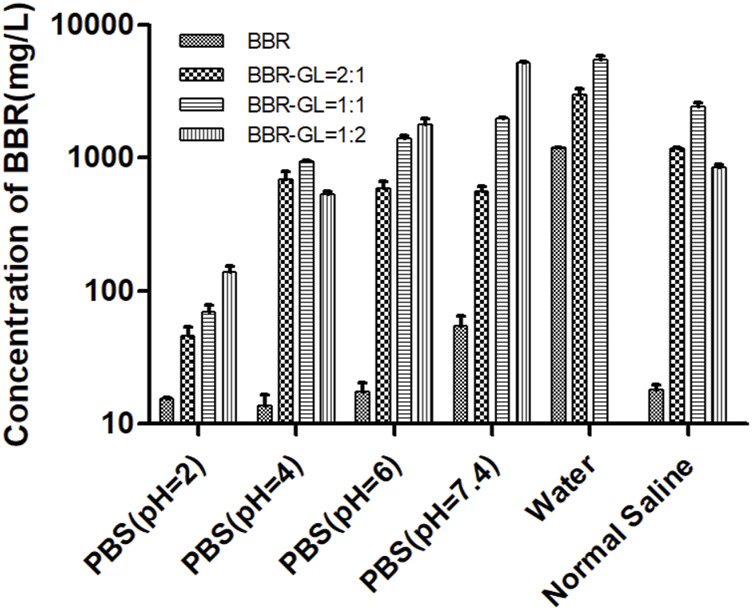
Solubility of berberine was determined by preparing the BBR-GL complex with different proportions in different pH solutions using HPLC. Data are presented as mean ± SD.

Figure 2 illustrated the solubility of BBR and BBR-GL complex in PBS with different pH (2, 4, 6, 7.4) solutions, pure water, and normal saline solution. The poor solubility of BBR in PBS and normal saline was less than 70 mg/l. As we expected, the solubility of BBR was dramatically increased in the existence of GL, which could be up to nearly 6,000 mg/l in PBS (pH 7.4) with the proportion of BBR and GL 1:2. Interestingly, the solubility of BBR in pure water was not as low as in PBS and normal saline. In this work, we used berberine chloride to perform a solubility assay. The hydrochloride of BBR was more soluble than BBR in water, but the solubility will decrease once the pH value and ionic strength changed in the solution. It is one of the reasons that BBR shows very poor aqueous solubility in biological fluids and other buffer solutions. Although the 1:2 proportion of BBR and GL exhibited the strongest solubilization effect, the complex form easily transformed into a gel state due to the high proportion of GL. On the other hand, the 1:1 proportion is compatible with TCM. Thus we used the proportion of 1:1 for our subsequent study. Similarly, the solubility of the 1:2 proportion of BBR and GL in water was not shown as it was more inclined to form a gel in water. To sum up, these results indicated that GL could increase the solubility of BBR in the proportion of 1:1 or 1:2. And we chose 1:1 for the follow-up study considering the properties of the BBR-GL complex.

### 3.2 Characterization of the Berberine–Glycyrrhizic Acid Complex

**FIGURE 3:**
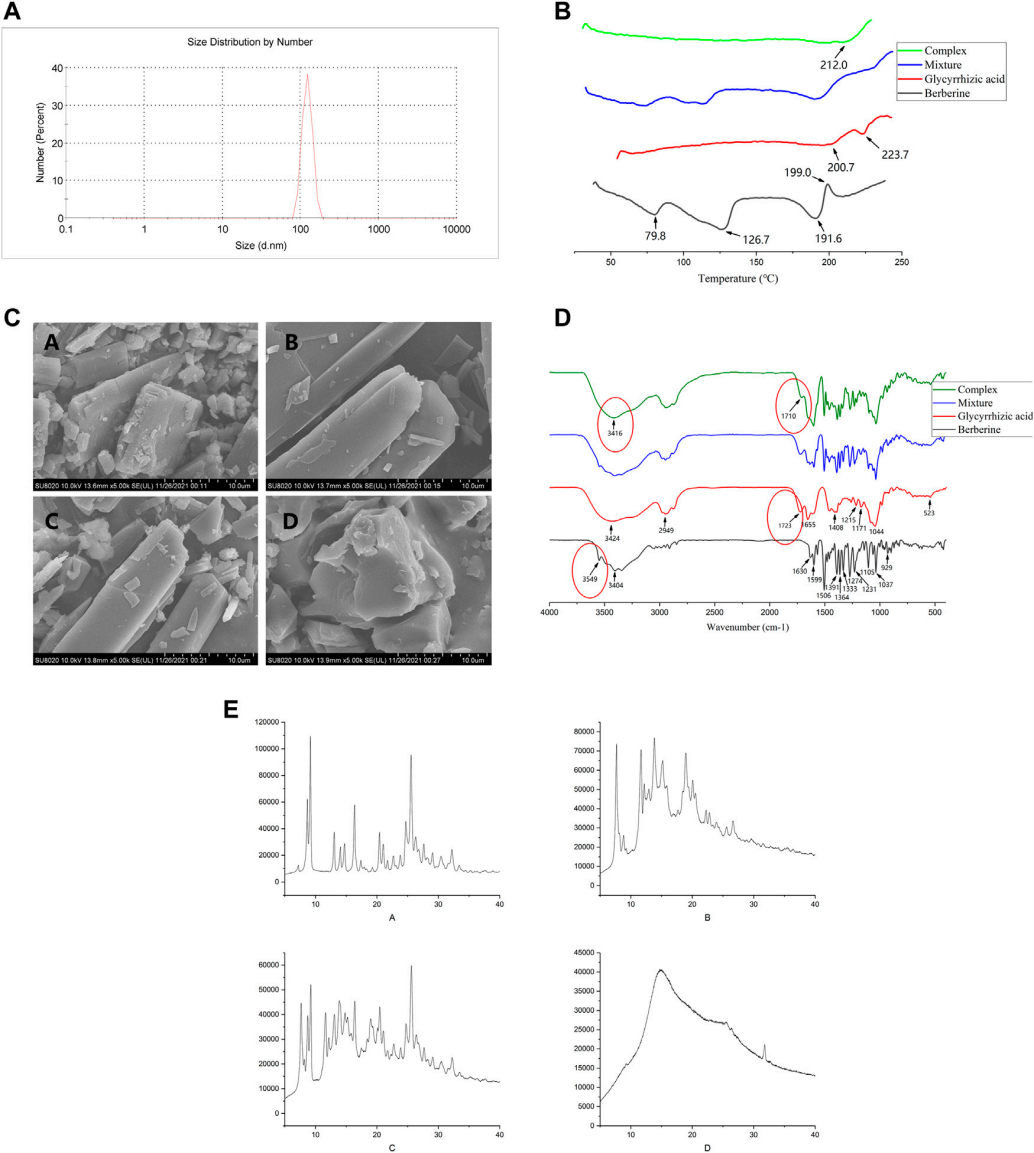
Characterization of the BBR-GL complex. **(A)**. Size distribution of BBR-GL complex. **(B)**. SEM of **(A)** BBR, **(B)** GL, **(C)** mixture, and **(D)** BBR-GL complex. **(C)**. DSC of **(A)** BBR, **(B)** GL, **(C)** mixture, and **(D)** BBR-GL complex. **(D)**. FT-IR of **(A)** BBR, **(B)** GL, **(C)** mixture, and **(D)** BBR-GL complex. **(E)**. PXRD of **(A)** BBR, **(B)** GL, **(C)** mixture, and **(D)** BBR-GL complex.

The morphology of BBR, GL, BBR/GL mixture, and BBR-GL complex was observed by SEM (Figure 3). BBR, GL, and the mixture showed an angular and rodlike appearance while the sphere-shaped surface of the complex was observed, which accounted for the encapsulation of GL in the course of self-assembly due to its unique amphiphilic structure. The size of the BBR-GL was 137.4 ± 2.4 nm.

In addition, to investigate the existing form of BBR in the BBR-GL complex, DSC analysis was conducted for BBR, GL, mixture, and BBR-GL complex with the same composition. The DSC thermogram of BBR and GL exhibited an endothermic peak at 199 and 223.7°C, respectively, which was attributed to their melting. In the BBR/GL mixture, endothermic peaks were observed as pure BBR (approximately 195°C) and pure GL (approximately 200 and 220°C), while the complex showed a neutral peak (212°C). These results suggested that the majority of the BBR was encapsulated by GL in the prepared BBR-GL complex.

The FT-IR spectra of BBR, GL, mixture, and complex also showed characteristic features. BBR showed absorption peaks at 3,404 cm^−1^, which was assigned to O–H stretching vibration, which could be explained by the presence of some possible water molecules that adhere to BBR crystals (Lu et al., 2019). The strong absorption bands of BBR at 1,630 cm^−1^, 1,599 cm^−1^, and 1,506 cm^−1^ were attributed to C=C stretching vibration. For the complex, the absorption peak at 3,549 cm^−1^ of BBR vanished, and the peaks at 3,416 cm^−1^ of O–H and 1,710 cm^−1^ of C=C appeared, which were different from the mixture. They could be accounted for the redshift of related functional groups during the course of the process.

The sample of the BBR-GL complex exhibited a unique PXRD pattern. New diffraction peaks appeared and characteristic peaks of the individual components were mostly absent. The BBR-GL complex showed a unique PXRD pattern with a flat curve, the latter part of which was entirety like GL and retained a characteristic peak of BBR at 33.5°. This suggested that a new complex form was produced during the course of the process.

### 3.3 *In Vitro* Drug Release of Berberine

The BBR solution and BBR/GL physical mixture showed similar *in vitro* drug release profiles with an initial burst release in water. The release from both BBR solution and mixture rapidly reached over 80% in the first hour and the drugs were almost completely released within 5 h. Obviously, GL showed no effect on the release of BBR when BBR was just physically mixed with GL, whereas the BBR-GL complex exhibited a sustained release pattern with <40% of BBR release in the first hour and the cumulative release amount under 80% at 5 h. But the cumulative release amount of BBR-GL complex gradually became close to BBR solution over 12 h. These notable distinctions of drug release profiles between the BBR/GL physical mixture and the BBR-GL complex confirmed that the structure of the BBR-GL complex could not be destroyed immediately under physiological condition, but the release of BBR from the complex would not take too long.

The *in vitro* drug release data of formulations were fitted in three different kinetic models (Table 1), that is, zero-order, first-order, and Higuchi models. The *in vitro* drug release from BBR, BBR/GL physical mixture, and BBR-GL complex was best fitted to first-order kinetic model as the plots showed the highest linearity (*r* ^2^ = 0.96, 0.92, and 0.95, respectively).

**TABLE 1:** The *in vitro* drug release kinetic models of BBR, BBR/GL physical mixture, and BBR-GL complex in different release media.

Model	BBR	*R* ^2^	BBR/GL mixture	*R* ^2^	BBR–GL complex	*R* ^2^
Zero order	*y* = 0.39*x* + 82.72	0.142	*y* = 0.51*x* + 77.99	0.21	*y* = 1.11*x* + 57.90	0.37
First order	*y* = 93.79(1–e^−3.08*x*^)	0.96	*y* = 92.19(1–e^−2.44*x*^)	0.92	*y* = 92.36(1–e^−0.61*x*^)	0.95
Higuchi	*y* = 3.81^1/2^ *x* + 76.58	0.33	*y* = 10.14^1/2^ *x* + 42.54	0.62	*y* = 4.93^1/2^ *x* + 70.15	0.42

### 3.4 Pharmacokinetics of Berberine and Its Major Metabolites in the Berberine–Glycyrrhizic Acid Complex

**FIGURE 4:**
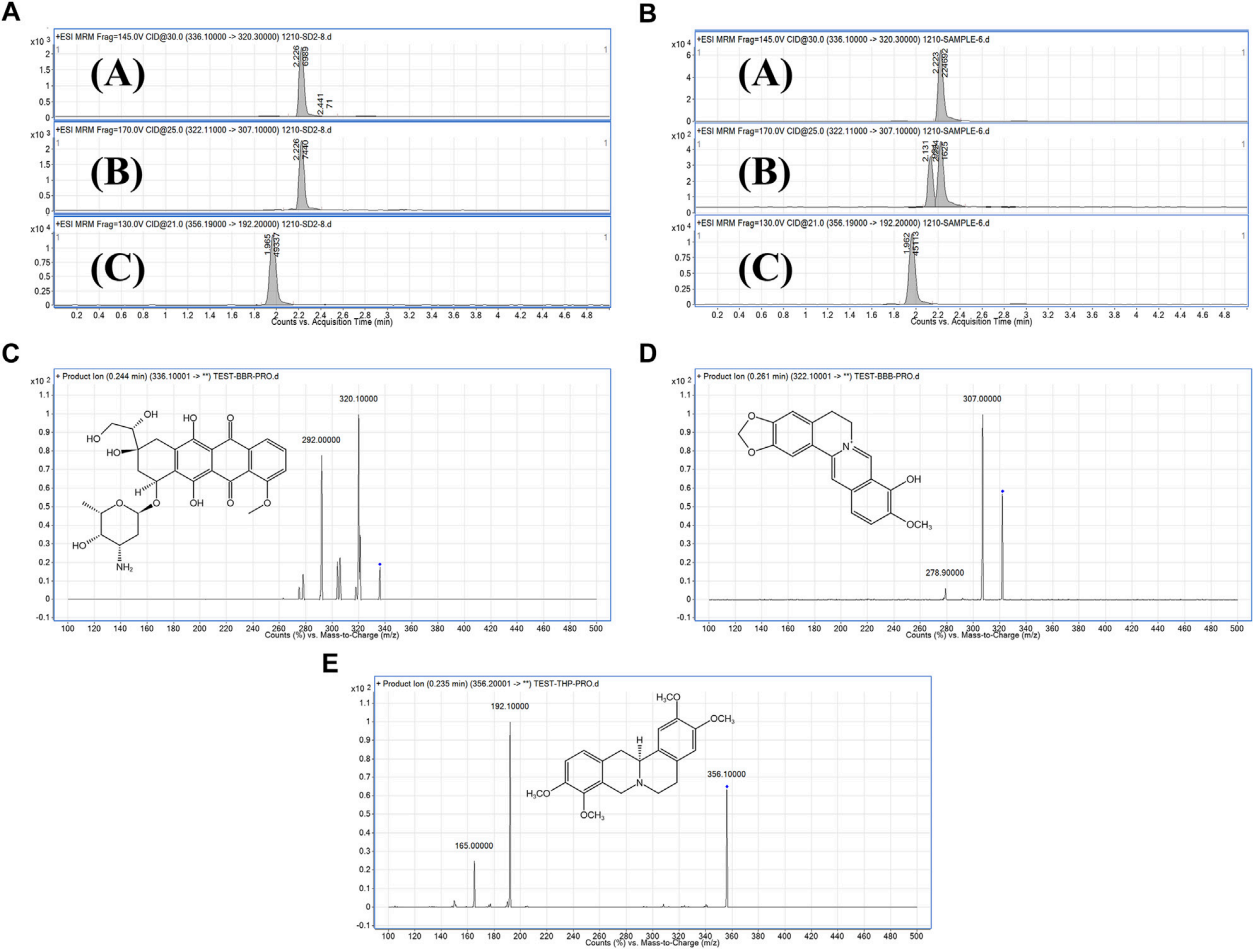
MRM chromatograms and secondary mass spectrometry chromatograms. **(A)**. Standard substance MRM chromatograms of **(A)** BBR, **(B)** BBB, and **(C)** IS. **(B)**. Sample MRM chromatograms of **(A)** BBR, **(B)** BBB, and **(C)** IS. **(C)**. Secondary mass spectrometry chromatograms of BBR. **(D)**. Secondary mass spectrometry chromatograms of BBB. **(E)**. Secondary mass spectrometry chromatograms of IS.

To investigate the pharmacokinetic characteristics of BBR and BBR-GL complex, the concentrations of BBR and the major metabolic BBB in plasma were determined by LC-MS/MS after a single dose of BBR and BBR-GL in male SD rats including an oral administration containing 40 mg/kg BBR solution and BBR-GL complex, as well as a single intravenous injection of BBR and BBR-GL containing 1 mg/kg BBR. As shown in Figure 4, the THP peaks of BBR, BBB, and IS compound were identified from the plasma samples using MRM mode. The fragmentation patterns of these peaks were identical to those from the authentic standard compounds. The LC-MS/MS method for pharmacokinetics showed good selectivity, high sensitivity, and fast analysis speed, which fit BBR and metabolic pharmacokinetic study.

**FIGURE 5:**
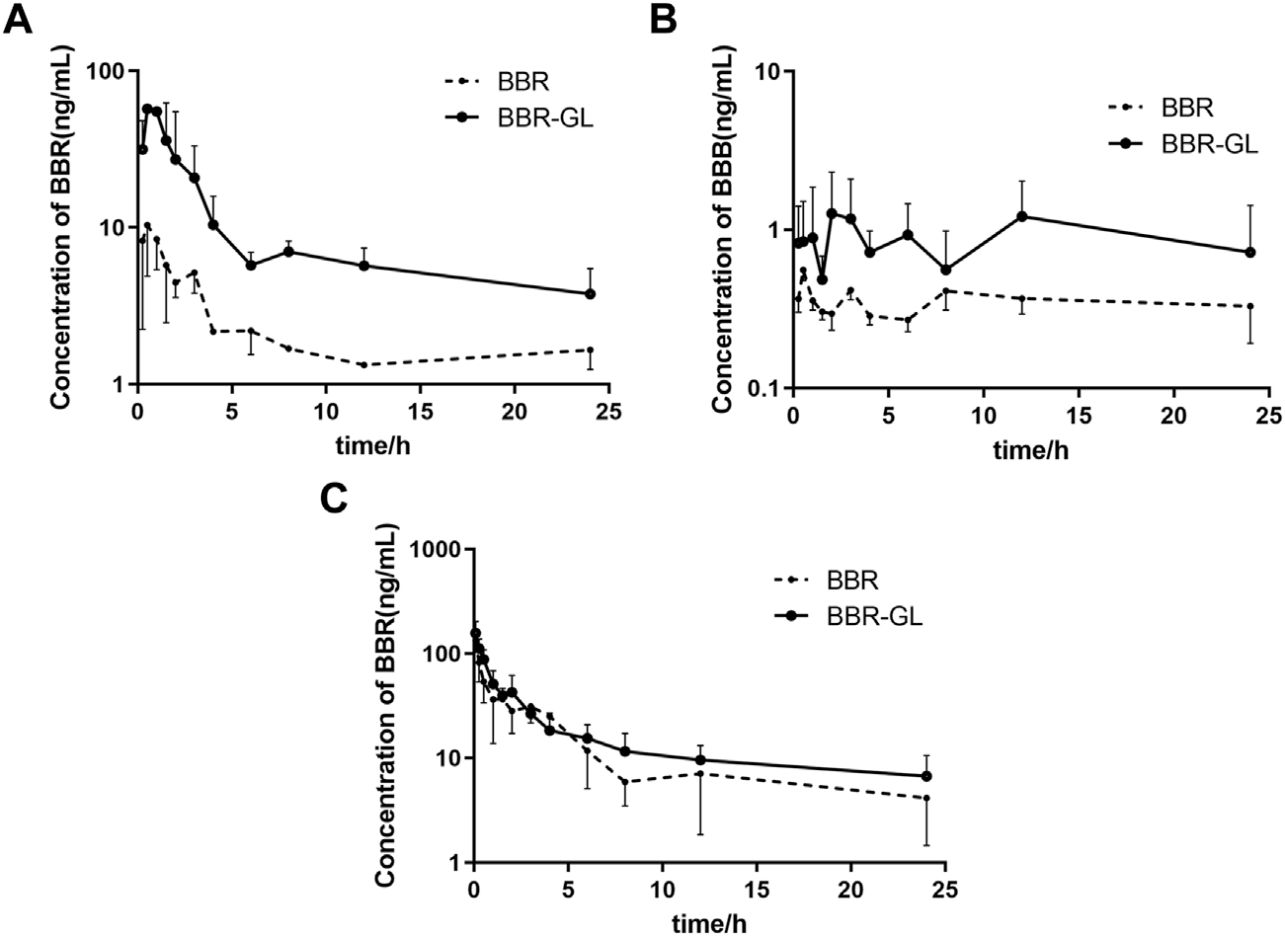
Mean plasma concentration–time curves in rats of **(A)** BBR, **(B)** BBB following oral administration, and **(C)** BBR following intravenous injection of BBR and BBR-GL. Data are presented as mean ± SD.

The mean plasma concentration−time curves in rats was shown in Figure 5. As shown in Table 2, the *T*_max_ of BBR in PO administration was 0.458 ± 0.292 h, which was similar to the other research (Kwon et al., 2020). Our pharmacokinetic work figured out that the BBR-GL complex significantly increased the BBR concentration in each sampling time in PO administration. The AUCs of the BBR-GL complex and BBR solution group were 201.615 ± 107.232 and 45.530 ± 17.007 ng h/ml, respectively (Tables 2, 3), though the AUCs of both BBR and the BBR-GL complex in injection administration had no significant difference. It meant that the solubilization effect in the BBR-GL complex formulation for BBR markedly elevated the absorption of BBR and increased the bioavailability by 4.43-folds (0.393 vs. 1.741%).

**TABLE 2:** Pharmacokinetic parameters of BBR and its metabolism following oral administration (40 mg/kg) and intravenous injection (1 mg/kg).

Administration	Unit	BBR (PO, 40 mg/kg)	BBB	BBR (Inj, 1 mg/kg)
Parameters		BBR		BBR
*T*_max_	h	0.458 ± 0.292	2.125 ± 3.057	
*C*_max_	ng/ml	12.532 ± 4.102	0.666 ± 0.096	133.711 ± 23.807
AUC_0–24_	ng·h/ml	45.530 ± 17.007	6.292 ± 3.009	289.484 ± 134.342
Metabolic ratio	%		13.819	
Bioavailability	%	0.393		

*T*_max_, time to reach maximum concentration; *C*_max_, maximum concentration; AUC_0–24_, area under the plasma concentration–time curve from time 0 to 24 h of quantifiable concentration. Data are presented as mean ± SD.

**TABLE 3:** Pharmacokinetic parameters of BBR–GL and its metabolism following oral administration (containing 40 mg/kg BBR) and intravenous injection (containing 1 mg/kg BBR).

Administration	Unit	BBR–GL (PO, 40 mg/kg)	BBB	BBR–GL (Inj, 1 mg/kg)
Parameters		BBR		BBR
*T*_max_	h	0.667 ± 0.492	6.333 ± 5.007	
*C*_max_	ng/ml	63.829 ± 50.383	1.947 ± 0.713	156.758 ± 45.200
AUC_0–24_	ng·h/ml	201.615 ± 107.232	23.733 ± 12.666	360.074 ± 88.264
Metabolic ratio	%		11.771	
Bioavailability	%	1.741		

*T*_max_, time to reach maximum concentration; *C*_max_, maximum concentration; AUC_0–24_, area under the plasma concentration–time curve from time 0 to 24 h of quantifiable concentration. Data are presented as mean ± SD.

Meanwhile, to evaluate the metabolism of BBR in the BBR-GL complex, we found the concentration and AUC of BBB were increased in the BBR-GL complex while the BBR concentration was promoted. The metabolic ratio of BBB, calculated by the AUC_0–24 h_ of BBB to BBR, showed similar results in BBR solution and the BBR-GL complex. Thus, we considered that the BBR-GL complex in oral administration could improve the absorption and bioavailability of BBR while the metabolism of BBR might not be significant inference *in vivo*.

### 3.5 Berberine–Glycyrrhizic Acid Complex Enhances the Protective Effect of Berberine and Glycyrrhizic Acid Against Acute Doxorubicin-Induced Cardiac Injury

**FIGURE 6:**
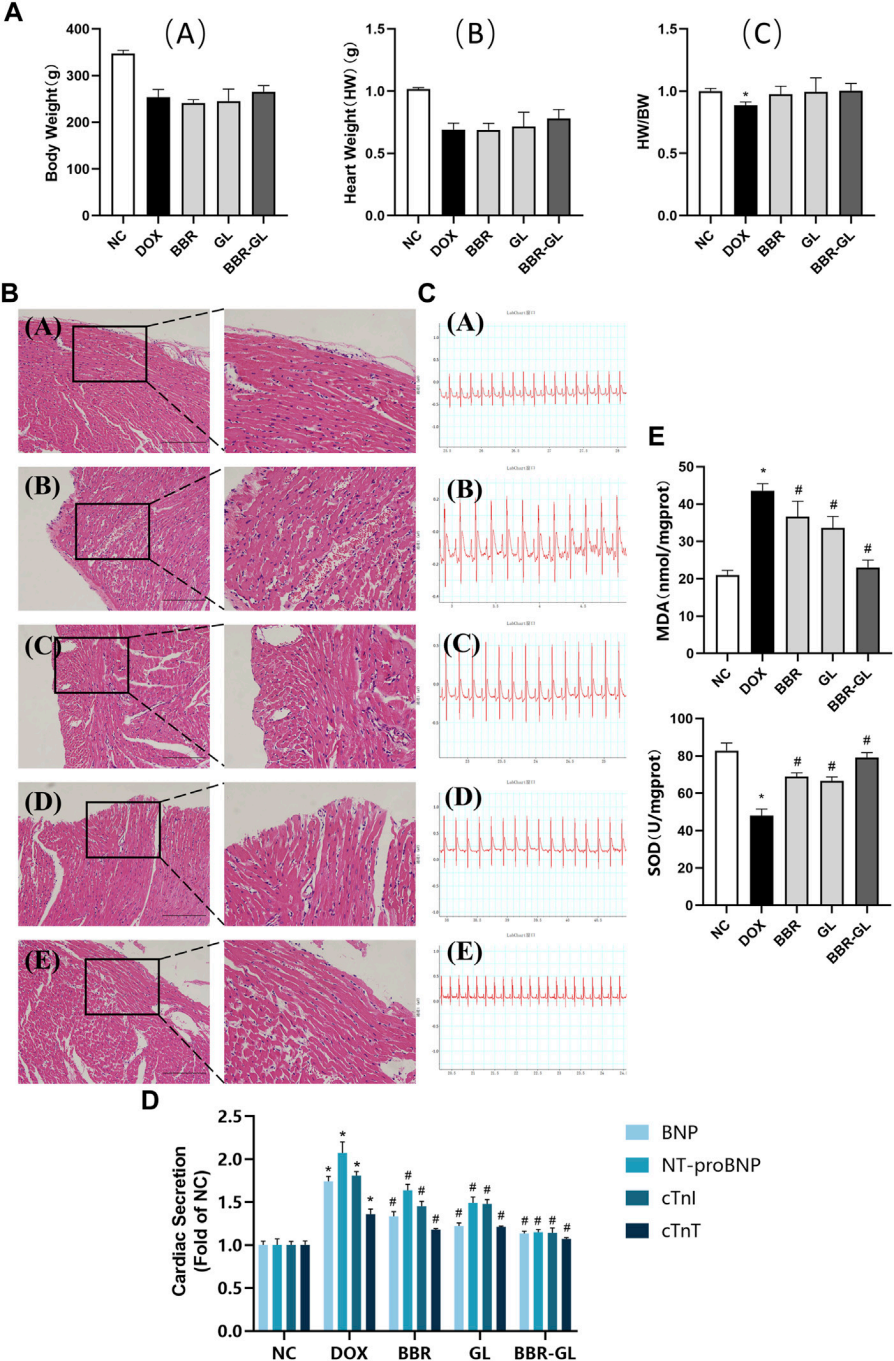
BBR-GL complex enhances the protective effect of BBR and GL against acute DOX-induced cardiac injury. **(A)**. Effects of DOX, BBR, GL, and their combination on the rat body weight and heart weight. **(A)** Body weight changes in rats; **(B)** heart weight changes in rats; and **(C)** changes of heart weight/body weight ratio. **(B)**. HE staining and partial magnification of **(A)** NC, **(B)** DOX, **(C)** BBR, **(D)** GL, and **(E)** BBR-GL. Scale bar: 100 μm. **(C)**. ECG of **(A)** NC, **(B)** DOX, **(C)** BBR, **(D)** GL, and **(E)** BBR-GL. **(D)**. Cardiac secretion of BNP, NT-proBNP, cTnI, and cTnT in rat plasma. **(E)**. MDA and SOD levels in rat heart. ^∗^ *p* < 0.05 vs. NC group; #*p* < 0.05 vs. DOX group. Data are presented as mean ± SD.

We first explored the possible toxicity or side effects of BBR-GL by hemolytic parameters and histopathological examinations. As shown in Table 4, all the parameters were within normal limits and no significance difference existed between the control and the single BBR-GL group, which indicated that 11-day BBR-GL treatment was innoxious for rats. Then we explored whether BBR and BBR-GL could protect rats from acute DOX-induced cardiac injury by body weight loss, heart to body weight ratio, and morphology. As shown in Figure 6, all groups modeling with DOX significantly lost body weight and heart weight, but the heart to body weight ratio was not changed markedly except in the DOX model group. The protective effect of BBR-GL was further verified by histopathological analysis (Figure 6). The heart tissue revealed myocardial cell injury, including capillary congestion, interstitial edema, and inflammation infusion, in different degrees when underexposed to DOX, whereas BBR, GL, and BBR-GL groups could attenuate the myocardial cell injury induced by DOX. The BBR-GL complex dramatically reduced the DOX-induced histopathological damage. A rat in the DOX-only-treated group (10%) died 1 day after the DOX administration. However, no mortality was observed in any of the other groups, including the combined BBR + DOX-treated group and the combined BBR-GL + DOX-treated group.

**TABLE 4:** Hemolytic parameters in CBC analysis of the blood and biochemical analyses of liver and kidney function indexes of the serum from NC- or BBR–GL-treated rats.

Parameters	NC	BBR–GL	*p* value
WBC (10^9^/l)	2.71 ± 0.54	2.42 ± 0.32	0.334
RBC (10^12^/l)	7.43 ± 0.68	6.84 ± 0.35	0.124
HGB (g/l)	144.2 ± 3.35	139.2 ± 3.7	0.055
HCT%	43.28 ± 0.95	42.48 ± 0.45	0.129
MCV (fl)	62.26 ± 2.84	60.98 ± 2.56	0.476
MCH (pg)	20.58 ± 0.75	20.38 ± 0.86	0.706
MCHC (g/l)	329.8 ± 4.82	334.0 ± 4.95	0.211
PLT (10^9^/l)	1,192 ± 56.83	1,189 ± 129.1	0.968
ALT (U/l)	33.4 ± 6.21	39.66 ± 1.55	0.060
AST (U/l)	98.04 ± 11.70	92.54 ± 5.37	0.367
TBIL (μmol/l)	0.90 ± 0.19	1.38 ± 0.29	0.063
UA (μmol/l)	96.40 ± 17.44	87.40 ± 14.74	0.404
Cr (μmol/l)	16.80 ± 3.03	17.40 ± 1.95	0.719
UREA (μmol/l)	4.60 ± 0.89	4.34 ± 0.69	0.620

WBC, white blood cell count; RBC, red blood cell count; HGB, hemoglobin. HCT, hematocrit; MCV, mean corpuscular volume; MCH, mean corpuscular hemoglobin. MCHC, mean corpuscular hemoglobin concentration; PLT, platelet count/blood platelet count; ALT, alanine aminotransferase. AST, aspartate aminotransferase; TBIL, total bilirubin; UA, uric acid. Cr, creatinine; UREA, urea formaldehyde. Data are presented as mean ± SD.

The influence of the treatment with BBR, BBR–GL complex, and/or DOX on the rat ECG parameters was shown in Figure 6 and Table 5. The ECG tracing showed the change in the DOX-treated group with the BBR–GL complex ameliorating this change. Compared with the negative control group, the heart rate of the DOX model group decreased and the QRS and QT intervals increased significantly. On the contrary, in the BBR and BBR–GL complex treatment group, compared with the DOX model group, the heart rate increased and QRS and QT intervals decreased. Especially in the BBR–GL complex group, the ECG parameters reverted to the normal level shown in the negative control group. To further examine the effects of BBR–GL against DIC in rat hearts, the release of BNP, NT-proBNP, cTnI, and cTnT, the four important biomarkers of heart injury, were measured. Figure 6 indicated that DOX treatment significantly increased the release of BNP, NT-proBNP, cTnI, and cTnT, but they were remarkably reduced by BBR, GL, and BBR–GL. Furthermore, we measured the MDA and SOD levels in heart tissue to evaluate the antioxidant effect in preventing DOX-induced acute myocardial toxicity. Figure 6 showed that the levels of MDA in the DOX model group were significantly adjusted compared with control, while BBR and GL reversed the change and the BBR–GL complex could downregulate the MDA level similar to the control group. In addition, the levels of SOD in the DOX group were remarkably downregulated, while the BBR-GL showed stronger effectiveness than BBR and GL to upregulate the SOD level in heart tissue, indicating that BBR-GL could restore the MDA and SOD levels in the presence of DOX.

**TABLE 5:** Influence of BBR and BBR–GL on ECG parameters of rats treated with DOX. *p < 0.05 vs. NC group; #*p* < 0.05 vs. DOX group.

Group	Heart rate (beat/min)	QRS interval (ms)	QT interval (ms)
NC	446.8 ± 10.0	18.3 ± 2.1	53.0 ± 3.6
DOX	373.8 ± 4.3^∗^	34.0 ± 1.3^∗^	72.0 ± 2.3^∗^
BBR	403.9 ± 4.3#	27.5 ± 1.4#	67.1 ± 2.7#
GL	412.5 ± 6.9#	27.5 ± 2.1#	64.8 ± 2.8#
BBR-GL	436.9 ± 9.5#	17.3 ± 0.8#	53.2 ± 0.6#

Data are presented as mean ± SD.

## 4 Discussion

Berberine has been used as a certain efficacy therapeutic agent of diarrhea and as antimicrobial for a long time in the wide-ranging world (Song et al., 2020). Berberine also has therapeutic potential as antidiabetic and antihyperlipidemic and has cardiac injury protective effects (Li DD. et al., 2020; Xu et al., 2020; Ding et al., 2021). However, berberine has not been widely used clinically because of its extremely low plasma concentrations and rapid elimination (Liu et al., 2016). Also, BBR injection is not used clinically as its safety has not been proved and first-pass elimination still exists. When administered orally, less than 1% of the common dose of berberine could penetrate the intestinal membrane into blood circulation. Considering the absorption in berberine pharmacokinetics, P-gp has been figured to play an important role in berberine efflux (Zamek-Gliszczynski et al., 2021). Therefore, to improve berberine bioavailability, there was a need to explore a new berberine delivery system to increase the solubility while inhibiting the P-gp efflux effect. However, as P-gp as well as CYP450 participated in a variety of drug absorption and transport, up- or downregulating the expression and function could lead to complex drug interactions, which may cause a serious adverse reaction. Thus, we considered constructing an oral berberine formulation that could not influence P-gp and CYP450 significantly while enhancing the bioavailability of berberine.

In this work, we developed a novel BBR-loaded complex formulation by using GL as a carrier, which has been found in our previous work to improve the solubility of paclitaxel (Yang et al., 2015), podophyllotoxin (Wang et al., 2016), and hydroxycamtothecin (Cai et al., 2019). We found this BBR-GL complex enhanced the solubility of BBR by over 100-fold in PBS (pH = 7.4). Although the proportion of BBR and GL at 1:2 and above showed a better effect in increasing BBR solubility, the solution of the complex easily transformed to gel form in both water and ion solution. Besides, the dose level of BBR in clinical use is quite high; so, many adverse effects might occur due to the high dose of GL. So we chose the 1:1 proportion of BBR-GL complex to be more compatible with TCM, for our pharmaceutics, pharmacokinetics, and pharmacological evaluation by considering druggability and safety application in clinic.

For the characterization of the BBR-GL complex, the DSC measurement gave an insight into the phase transition in the BBR-GL complex, which was responsible for the change in the physical state. The result of DSC clearly suggested that BBR was partly encapsulated by GL in the complex. This finding was further confirmed by the SEM assay, which showed the sphere-shaped surface of the complex, accounting for the encapsulation of GL in the course of self-assembly due to its unique amphiphilic structure. The infrared spectra of berberine, glycyrrhizic acid, the physical mixture, and the complex also showed relevant characteristics. The BBR-GL complex showed a different absorption peak from those of the BBR/GL physical mixture. This could explain the red shift of related functional groups during the formation of the complex. Additionally, the BBR-GL complex exhibited a unique PXRD pattern. New diffraction peaks appeared and characteristic peaks of the individual components were mostly absent. This suggested that a new complex form was produced in the course of the process. Hence, the BBR-GL complex is a novel form of BBR formulation, which is quite different from the BBR/GL physical mixture in chemical characterization. Likewise, the size of the BBR-GL complex was less than 200 nm, which prevented it from being captured by reticuloendothelial phagocytes (Choi et al., 2014) and was available to make itself permeate into the intracellular area.

**FIGURE 7:**
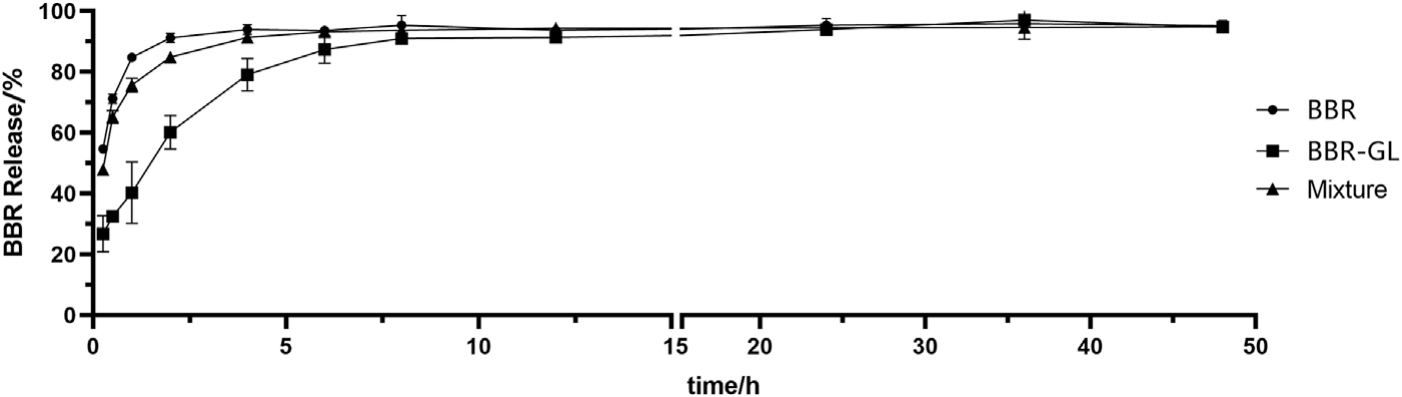
The *in vitro* drug release curve of BBR, BBR/GL mixture, and BBR-GL complex in pure water. Data are presented as mean ± SD.

BBR exhibits poor aqueous solubility in ion solution and acidic environment. In our study, BBR and BBR-GL complex showed different release curves *in vitro*, indicating the BBR-GL complex changed the release of BBR. We used water (Figure 7) and PBS (pH = 7.4) (data not shown) as the release medium and found quite a different release manner of BBR and BBR-GL complex in water and PBS. The release degree of BBR and BBR-GL complex solution reached more than 90% after 12 h, with BBR releasing faster than BBR-GL complex in water. But the release curve in PBS showed only 50% final release of BBR and BBR-GL complex, which might be caused by the lower solubility of BBR in PBS than in pure water.

BBR could undergo extensive first-pass metabolism in both intestine and liver, which resulted in a secondary plasma peak due to the two maximum concentrations observed at 1 and 8 h, respectively. Interestingly, enterohepatic circulation of berberine metabolite BBB was seen in our study, which was also reported by Zuo et al. (2006).

Consistent with the reported results (Chen et al., 2011; Feng et al., 2020), the absolute bioavailability of BBR was found to be 0.393%, which was greatly promoted to 1.741% of BBR-GL complex in PO administration. The AUC of the BBR-GL complex in PO administration was 4.4-folds bigger than that of the BBR solution group (201.615 ± 107.232 vs. 45.530 ± 17.007 ng h/ml), although the AUCs of both BBR and BBR-GL complex in injection administration had no significant difference. The above results indicated that the BBR-GL complex in oral administration could improve the absorption and bioavailability of BBR, suggesting that BBR-GL may play a role through the gastrointestinal tract rather than blood. The possible mechanism may be that BBR-GL can turn into a compound that promotes or exhibits the related substrate of transporter, in the specific pH of gastrointestinal tract, while the pathway is not available as a complete form in the blood. We also compared the pharmacokinetic differences after intravenous injection of BBR and BBR-GL, but no significance was found, which indicated that the BBR-GL might exert a special effect orally.

The metabolism of berberine in rats had been widely reported (Wang et al., 2017). Herein we chose BBB, the most abundant phase I metabolite of BBR, for the pharmacokinetic study. Our results showed that BBB was increased in the BBR–GL complex while the BBR concentration was promoted. The metabolic ratio of BBB, calculated by AUC_0–24 h_ of BBB to BBR, showed similar results in BBR solution and BBR-GL complex, which indicated that the metabolism of BBR might not be significant *in vivo*.

Many researchers has used PEG (Sujitha et al., 2020), liposome (Allijn et al., 2017), TCM extracts (Zhao et al., 2021), cremochylomicrons (Elsheikh et al., 2018), and pluronic P85 and tween 80 (Kwon et al., 2020) to improve the solubility and bioavailability of BBR. However, there is not enough evidence to prove these ingredients or extract meet the requirements of safety and stability. In contrast, GL, a natural extract from licorice, has been proved to be nontoxic in conventional dose and low poison in ultra heavy dose (>610 mg/kg) (Cosmetic Ingredient Review Expert Panel, 2007; Ming and Yin, 2013). Clinically, a dose of compound glycyrrhizin tablets contains about 315 mg/kg monoammonium glycyrrhizinate. The dose of BBR-GL used in our research contained 40 mg/kg BBR, which was converted to 65 mg/kg GL according to the molecular ratio, far less than the possible poisonous dose or clinical dose, proving that the dose of the complex is reasonable and safe in our research. Furthermore, the way to use drug to cover with drug could avoid the unknown risk of ingredients and increase the compatibility of drug. The steroid-like structure of GL increases not only the solubility of lyophobic drugs, but also their penetration through cell membranes by increasing the permeability (about 60%). Meanwhile GL acts as an anti-inflammatory to reduce the possible negative stimulation of the encapsulated drug (Selyutina et al., 2016). Interestingly, it was demonstrated that the application of GL together with some medicines could strengthen their therapeutic actions (Selyutina et al., 2016) and reduce their side effects.

Hemolytic parameters, such as various blood cell counts and hemoglobin level, and histopathological examinations, such as liver function indexes and kidney function indexes, are important parameters for safety aspects of any formulation. So we tested the possible toxicity or side effects of BBR-GL. BBR-GL had no obvious hematotoxicity, hepatotoxicity, and nephrotoxicity as indicated by the hemolytic parameters and biochemical indexes of liver and renal function measured from the blood and the serum of rats after 11-day BBR-GL treatment. The parameters in the BBR-GL group were in the normal range. BBR-GL could be proved to be safe with no significant difference found between the two groups, although ALT and TBIL showed comparatively more variation, which may be because of the metabolism of BBR-GL in the liver.

Acute cardiotoxicity usually occurs in the initial stage of drug use and usually lasts for a short time and can be reversed. Therefore, it is of great significance to study the acute cardiotoxicity of DOX to reduce its side effects and increase its anticancer efficacy. *In vivo* DIC model was constructed by single-dose DOX (20 mg/kg, ip) as per other literatures (McCluskey et al., 2019; Lv et al., 2020; Mohammed et al., 2020). The acute DIC was manifested by the abnormal change of ECG, with reversible cardiac dysfunction sometimes. In our study, the heart rate descended with QT intervals and QRS intervals increasing after DOX administration, indicating that DOX successfully caused significant myocardial damage and reduced ventricular function (bradycardia), which was also illustrated by HE staining. However, this destructive effect could be reversed by BBR, GL, and BBR–GL, improving the ECG parameter and cardiac histopathological morphology. In addition, the BBR, GL, and BBR-GL treatment significantly alleviated the DOX-induced global redox injury in rats by increasing the antioxidant enzyme SOD and significantly reducing the cardiac secretion of BNP, NT-proBNP, cTnI, and cTnT as well as the levels of heart lipid hydroperoxide marker MDA, with the effect of BBR-GL more obvious. In our previous study (Lv et al., 2020), we found GL improved autophagy flux *via* HMGB1-dependent Akt/mTOR signaling pathway and contributed to attenuating DIC. On the contrary, BBR could ameliorate DIC *via* an SIRT1/p66Shc-mediated pathway according to a study (Wu et al., 2019). In BBR-GL administration, GL promoted the bioavailability of BBR, which helped BBR–GL to display more therapeutic action for DIC than BBR.

Although the BBR–GL complex exhibited an applicable property *in vitro* and *in vivo*, our work clearly had some limitations. The existing work in our research could not confirm the concrete form of BBR-GL on a molecular level. In addition, further research on the increase in solubility of BBR in BBR–GL is needed. The long-term stability of BBR–GL was not figured out in our study. though the safety was proved.

To sum up, our novel BBR-loaded BBR–GL complex formulation could increase BBR plasma concentration by elevating the solubility of BBR to increase the absorption in oral administration. Improvement of BBR bioavailability by BBR–GL complex could coordinate with GL to attenuate DOX cardiotoxicity. This could contribute to the beneficial effect of BBR–GL complex therapy in preventing cardiac damage in DOX clinical application.

## Data Availability

The raw data supporting the conclusion of this article will be made available by the authors, without undue reservation.

## Abbreviations

BBR, berberine; GL, glycyrrhizic acid; BBR-GL, berberine–glycyrrhizic acid; BBB, berberrubine; DOX, doxorubicin; DIC, doxorubicin-induced cardiotoxicity; FT-IR, Fourier transform infrared spectroscopy; DSC, differential scanning calorimetry; PXRD, powder X-ray diffractometry; IS, internal standard; ECG, electrocardiography; CBC, complete blood count; ALT, alanine aminotransferase; AST, aspartate aminotransferase; TBIL, bilirubin total; UA, uric acid; UREA, urea formaldehyde; CRE, creatinine; HE, hematoxylin and eosin; TCM, traditional Chinese medicine; AUC, area under the curve; HW, heart weight; BW, body weight; MDA, malondialdehyde; SOD, superoxide dismutase.
